# Searching for predictors of sense of quality of health: A study using neural networks on a sample of perimenopausal women

**DOI:** 10.1371/journal.pone.0200129

**Published:** 2019-01-03

**Authors:** Małgorzata Włodarczyk, Grażyna Dolińska-Zygmunt

**Affiliations:** Institute of Psychology, University of Wrocław, Wrocław, Poland; Universidad Nacional Autonoma de Mexico, MEXICO

## Abstract

**Background:**

We assumed that perimenopausal women’s sense of quality of health (SQH) is a subjective evaluation of their psycho-physical health, and comprises three dimensions: sense of quality of life, menopausal symptoms, and the level of positive and negative affect.

**Purpose:**

The aim of the study was to: 1) test a model about SQH, and 2) explore the role of personality traits, self-esteem, body self, and self-stereotype as predictors of SQH.

**Methods:**

The sample included 201 women aged between 45 and 55 (50.11±3.07). Participants filled out the Rosenberg Self-Esteem Scale, the Personality Inventory based on the Big Five Factor Model, the Body Self Questionnaire, and a survey querying perimenopausal women’s self-stereotype. To determine the individual SQH dimensions we used the Sense of Quality of Life Questionnaire, the Menopause Symptom List, and the Positive and Negative Affect Schedule. To verify the assumptions of the SQH model and look for SQH predictors we conducted a neural networks analysis with structure optimization via genetic algorithms (a multivariate analysis).

**Results:**

The SQH model was verified in the course of several neural networks analyses with structure optimization via genetic algorithms (R = 0.849, R^2^ = 0.723, F = 133,232, *p* < 0.01). Moreover, we confirmed that SQH comprised three dimensions: quality of life, menopausal symptoms, and affect. SQH and menopausal symptoms were correlated. Similarly, positive and negative affect modified the women’s global sense of quality of life. SQH predictors included: personality traits, self-esteem, the body-self, and menopausal woman’s self-stereotype.

**Conclusion:**

In practical terms, our findings may help raise awareness among women and medical practitioners, calling for a holistic approach to the health of menopausal women. Our findings may also facilitate the creation of both prevention and therapeutic programs for women transitioning through menopause, for example, cognitive-behavioral therapy.

## Introduction

Perimenopause is a neuroendocrine stage of woman’s life marked by changes affecting the somatic and psychosocial spheres of functioning, during which women confront social expectations about their health and vitality [1,2].

Menopausal women’s health has so far been analyzed in the context of experienced symptoms and occurring diseases [3,4], women’s socio-economic status and occupation, eating habits, use of stimulants, and physical activity [5]. The last decade has seen an increase in research aiming to identify the determinants of menopausal women’s health also among psychological, social, and cultural factors, including state of health and global sense of well-being, which is correlated with body image, among others [4,6–13].

Women’s well-being depends on numerous factors, including experienced stress, bodily and physical changes, experienced affect, thoughts, beliefs, behavioral strategies, and social and cultural conditions [2,6,14–23]. Thus, the predictors of menopausal women’s health should be sought by taking account of the subjective evaluation of their psycho-physical health, rather than merely relying on the tenets of the biomedical model [23].

Research confirms that menopausal experience is determined not only by biological factors (in particular those related to estrogen levels in the blood), but also social and cultural ones, including attitudes toward menopause held by society and the menopausal women themselves [6,9,22–23]. Attention is also given to psychological determinants, such as personality traits, self-esteem, and affect [2,14,16,19,24–26].

There are two models that emphasize the non-biological aspect of experiencing the menopausal period.

The first, cognitive behavioral model of menopausal hot flushes proposed by Hunter and Rendall, is focused on the relationships between the experienced hot flushes and decreased levels of mood and self-esteem, which imply a negative perception of menopausal symptoms (stronger and more frequent flushes) [6,14,16]. This model also underlines the biological determinants of menopause (such as hormonal changes and health condition), along with cultural determinants (beliefs, customs, stereotypes), psychological factors (self-esteem, earlier experiences, mood, the way of perceiving one’s body), and culturally-determined beliefs and attitudes toward menopause existing in social awareness [6,10,14,22,26].

The second model, by Veeninga and Kraaimaat, is multifactorial and refers to the development of bodily experiences and mood states during the climacteric, suggesting that the experience of menopause depends on the very process of perceiving and assessing menopausal symptoms [15]. The process can be modified as a consequence of focusing on the symptoms from the body in the context of the woman’s earlier experiences and social and individual expectations, which affect the way how perimenopausal women experience their bodies [14,26]. Negative attitude towards menopause was observed to imply the appearance of negative emotions, which had a detrimental effect on the experience of the menopausal period [6,10,22,26]. Moreover, somatic symptoms of menopause may contribute to an unstable body image, and lead to a sense of losing control over one’s body [14].

Despite the multiple research focused on the determinants of perimenopausal women’s health, there is a paucity of data on the predictors of the factors determining the sense of quality of health (SQH) in women around menopause.

Going beyond the assumptions of the biomedical model, we posited that SQH is a cognitive-affective subjective evaluation of psycho-physical health, comprising three dimensions: sense of quality of life (defined as a subjective evaluation of one’s life, which includes four spheres: biological, social, subjective, and metaphysical [27–31], menopausal symptoms (including the frequency and intensity of psychological, vasomotor, and somatic symptoms [32–33]), and positive and negative affect [34].

Based on literature review, we assumed that perimenopausal women’s SQH has three dimensions and depends on four variables: personality traits, self-esteem, body self, and self-stereotype [2,4–6,9,10–12,14–15,20,22–26].

Personality traits can be analyzed according to the Big Five Factor Model by Costa and McCrae [35]. Rosenberg’s approach can serve as a framework for the assessment of global self-esteem [36].

Within the concept of the body self, which is derived from the psychodynamic paradigm, additionally comprises attitude toward the body (body image), comfort with closeness with others, and body protection, issues pertaining to the development, structure and way of defining the body self, as well as its importance for human personality, can be defined using Krueger’s description [37–40].

Self-stereotype can be defined as the simplified and evaluative image of reality that functions in the popular awareness of perimenopausal women [41–44]. It pertains to how the woman looks and behaves, what she experiences, and how she defines the period of menopause.

### Aims and hypotheses

**Fig 1:**
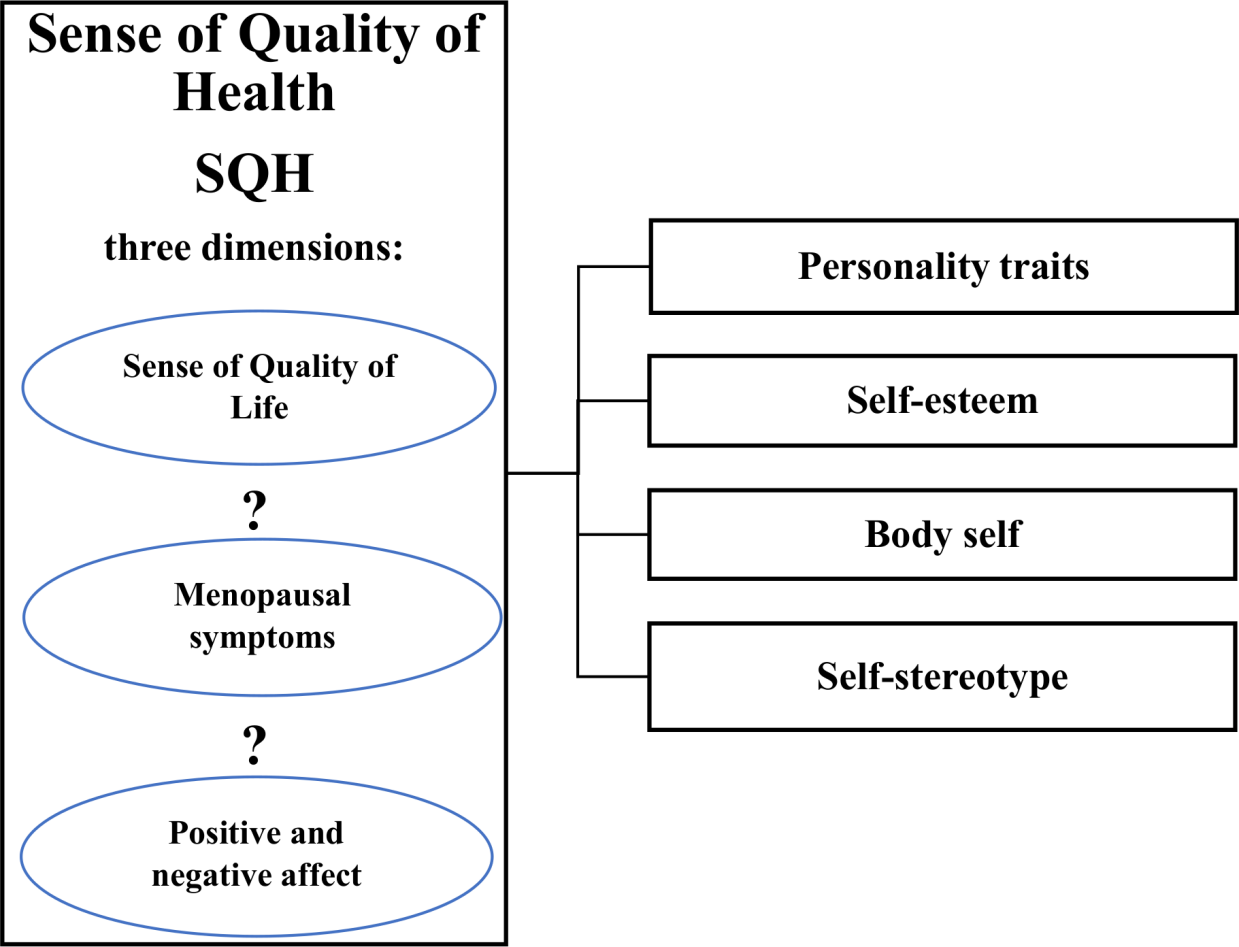
Proposed model of perimenopausal women’s sense of quality of health.

In sum, we propose that the sense of the quality of health should not be limited to the symptoms of menopause but also include the sense of quality of life and positive and negative affect (Fig 1).

Thus, the aims of the study were as follows:

1. To verify whether SQH comprises three dimensions: sense of the quality of life, symptoms of menopause, and positive and negative affect;
2. To identify the predictors of SQH among: personality traits, self-esteem, body self, and self-stereotype.

We decided to verify the following hypotheses:

H1: SQH comprises three dimensions: sense of the quality of life, frequency and intensity of menopausal symptoms, and positive and negative affect.
H2: Higher level of the sense of quality of life (along with its psycho-physical, psycho-social, subjective, and metaphysical spheres) is associated with higher levels of extraversion, openness, agreeableness, conscientiousness, and self-esteem, lower levels of neuroticism and a lesser dysfunction of the body-self (lower score), and a positive self-stereotype of the menopausal woman.
H3: Less frequent and less intense psychological, vasomotor and somatic symptoms of menopause are associated with higher levels of extraversion, openness, agreeableness, conscientiousness, and self-esteem, lower levels of neuroticism and a lesser dysfunction of the body-self (lower score), and a positive self-stereotype of the menopausal woman.
H4: Higher positive affect is associated with higher levels of extraversion, openness, agreeableness, conscientiousness, and self-esteem, lower levels of neuroticism and a lesser dysfunction of the body-self (lower score), and a positive self-stereotype of the menopausal woman.
H5: Higher negative affect is associated with lower levels of extraversion, openness, agreeableness, conscientiousness, and self-esteem, higher levels of neuroticism and a greater dysfunction of the body-self (lower score), and a negative self-stereotype of the menopausal woman

## Methods

### Participants

Participants were 201 married women aged 45–55 with a university degree in pedagogy, working as teachers in several schools in Wrocław, Poland. The main criterion for choosing this group was participants’ availability. Women using hormonal replacement therapy and those who underwent ovariectomy/hysterectomy leading to artificial menopause were excluded from the study. This information was obtained based on a survey attached to the questionnaires (see S1 File: Appendix A Menopausal status survey).

Participants’ menopausal status was determined using a survey developed for the purpose of this study, which was based on the Staging System for Reproductive Aging in Workshop (STRAW) (stages of reproductive and post-reproductive periods of women’s life developed to optimize research on the physiology and pathology of perimenopausal period).

Inclusion criteria: regularity and duration of the menstrual cycle, as well as the time and cause why menstruation ceased.

Exclusion criteria: women using hormonal replacement therapy and those whose medical treatment led to artificial menopause were excluded from the study.

This research project received approval of the Research Ethics Committee at the Psychology Institute, University of Wrocław. Participants were informed about the voluntary participation, the anonymous character of study (data was collected in a way that made it impossible to associate questionnaire answers with a specific participant), the aim and scope of the study, as well as the possibility of withdrawing at any stage. Before the questionnaires were distributed, we made sure that each participant was aware of what areas were covered by their consent to the study and understood the scope of information to be obtained and the intended use of the data to be collected. To minimize the negative impact the study could have on participant’s mental state, we offered the women psychological consultation after the completion of the study. Oral consent to the study was obtained from 201 participants.

### Questionnaires

We administered six questionnaires and two surveys: self-stereotype and a survey determining participants’ menopausal status and the time and cause why menstruation ceased. Participants filled them out in any given order.

#### 1. Measurement of SQH dimensions

**1.1. Measurement of sense of quality of life**

We used the Sense of Quality of Life Questionnaire in the Polish adaptation by Straś-Romanowska, Oleszkowicz, and Frąckowiak to measure participants’ level of this variable, a choice we made for two reasons [27–28]. First, we wanted to emphasize the subjective evaluation of participants’ sense of quality of life. Second, it is the only method measuring subjective sense of quality of life available to Polish psychologists. Using a method crated and developed abroad would make it necessary to adapt it to the Polish context, making the entire study a much more time-consuming endeavor.

The questionnaire consists of 60 statements, with 15 statements for each of the 4 spheres of well-being: metaphysical, psychosocial, subjective, and psycho-physical. For the purpose of this study, we calculated the normal values for perimenopausal women, including both a general score and individual scores for each of these four spheres. The standard scores were then converted to T-scale scores. The reliability of the instrument was as follows: for the general quality of life score–*α* = 0.70; for the psycho-physical sphere–*α* = 0.77; for the psychosocial sphere–*α* = 0.71; for the subjective sphere–*α* = 0.72; and for the metaphysical sphere–*α* = 0.65. The higher the score, the higher the sense of quality of life (see S2 File: Appendix B Measurement of quality of life).

**1.2. Measurement of frequency and intensity of menopausal symptoms**

The frequency and intensity of symptoms of menopause were determined using Perz’s Menopause Symptom List in the Polish adaptation by Bielawska-Batorowicz [32,33]. The symptoms included:

- psychological–irritability, depressed feelings, excitability, tense feelings, moodiness, crying spells, worrying needlessly, poor concentration, and pressure or tightness in head or body;
- vasomotor–numbness and tingling, loss of feeling in hands or feet, hot flushes, involuntary sweating, poor appetite, shortness of breath, palpitations, dry eyes, and cold hands and feet; and
- somatic–weight gain, dyspareunia, sleeplessness, loss of sexual interest, early morning awakenings, constipation, headaches.

Participants rate each of the symptoms on a 6-point scale, taking account of the frequency and intensity with which the symptom has occurred in the past three months. When rating symptom frequency, participants use the following scale: *Never* (0)–not even once in the past 3 months; *Rarely* (1)–once or twice in the past 3 months; *Sometimes* (2)–up to 4 times in the past 3 months; *Often* (3)–between 5 and 10 times; *Very often* (4)–more than 10 times in the past 3 months; *Almost always* (5)–almost every day in the past 3 months. In turn, the following 6-point scale is used to rate symptom intensity: (0)–the sensation did not occur; *Slight* (1)–a barely noticeable change or sensation; *Low* (2)–a small change, a weak sensation; *Moderate* (3)–a noticeable change / sensation; *High* (4)–a clearly noticeable change and an intense sensation; *Very high* (5)–a radical change and an extremely intense sensation. The raw scores are converted into sten scores.

The tool’s reliability, which ranged between 0.51 and 0.92, was calculated using the test-retest method. Its validity, which ranged between 0.39 and 0.89; *p* < 0.01 or *p* < 0.05, was determined based on correlations with the results of Green’s Climacteric Symptom Rating Scale. The higher the score, the higher the frequency and intensity of menopausal symptoms. Reliability of the Polish questionnaire was calculated using the test-retest method and by comparing parts of the test (Spearman-Brown coefficient). Cronbach’s alphas for the 3 subscales fell into the range between 0.70 and 0.87. In turn, correlation coefficients for test-retest ranged between 0.69 and 0.90, and were statistically significant (*p* < 0.01). High Cronbach’s alphas suggest satisfactory internal consistency of the instrument’s subscales, its high reliability additionally demonstrated by the Spearman-Brown coefficient (0.91 and alpha coefficients calculated for parts I and II of the test (0.85 and 0.84, respectively) (S3 File: Appendix C Measurement of menopausal symptoms and S9 File: Key My Measurement of menopausal symptoms)).

**1.3. Measurement of intensity of positive and negative affect**

To assess participants’ levels of positive (PA) and negative affect (NA), we used Watson and Clark’s (1988) Positive and Negative Affect Schedule (PANAS) in its Polish adaptation by Brzozowski (in Polish: *Skala Uczuć Pozytywnych i Negatywnych*; *SUPIN*) [34]. In this study, we used an abridged version of the C–20 scale, which measures the intensity of positive and negative affective traits. Participants were to assess the intensity of affects on a 5-point scale, ranging from 1 (*slight or none*) to 5 (*very strong*). The raw scores were then converted into sten scores. The instrument’s reliability (alpha coefficients) ranged between 0.73 and 0.95, depending on the version and type of sample. The higher the score, the higher the positive and negative affect (see S8 File: PANAS English version).

#### 2. Measurement of SQH determinants

**2.1. Measurement of personality traits**

To assess participants’ personality traits, we used Costa and McCrae’s NEO Personality Inventory (NEO PI-R) in the Polish adaptation by Siuta [45]. The tool consists of five factors: neuroticism, extraversion, openness, agreeableness, and conscientiousness, and a total of 240 statements. Participants respond to each statement on a 5-point scale, where A stands for *Strongly disagree*, and E for *Strongly agree*. To obtain raw scores, the scores for particular factors are summed and converted into sten scores. The instrument’s studies yielded a high degree of internal consistency for each of the five scales, ranging between *α* = 0.81 and 0.86. Factorial validity was also assessed, confirming that the subscales’ factor structure confirmed theoretical expectations, although some deviations from these assumptions were also found. The higher the score, the higher the level of each individual personality trait.

**2.2. Measurement of self-esteem**

Participants’ self-esteem levels were determined using the Rosenberg Self-Esteem Scale (RSES) in the Polish adaptation by Lachowicz-Tabaczek, Dzwonkowska, and Łaguna [36]. Designed to measure individuals’ global self-esteem, the scale consists of 10 statements to which participants respond on a 4-point scale, ranging from 1 (*Strongly agree*) to 4 (*Strongly disagree*). The result is the sum of points representing global self-esteem level. The raw scores are converted into sten scores. The scale’s reliability ranged between *α* = 0.81 and 0.83 for different age groups. The higher the score, the higher the participant’s self-esteem.

**2.3. Measurement of the body self**

To measure the body self, we used the Body Self Questionnaire developed by Sakson-Obada [39]. Created in the Polish context, the questionnaire has its theoretical underpinnings in the assumptions proposed by Krueger in *Integrating body self and psychological self*. *Creating a new story in psychoanalysis and psychotherapy* [38], which offer a detailed description of the role the body self plays in human psychological functioning. The instrument allows for measuring the capacity of the body self to receive bodily sensations (the strength of the body self), body image, comfort with closeness with others, and body protection. Participants respond to each statement on a 5-point scale, where 1 stands for *Never*; 2 –*Very rarely* (once or twice in a lifetime); 3 –*Sometimes*; 4 –*Quite often*; and 5 –*Very often*. The measure of the above mentioned scales is the sum of scores obtained on a given scale, which are then averaged so that the participant’s score falls within the range <1,5> [39]. The higher the averaged score, the higher the degree of disorder the participant exhibits within a given scale. This principle applies to all the scales making up the Body-Self Questionnaire.

Reliability assessed for particular scales of the questionnaire ranged between *α* = 0.62 and 0.89 (S4 File: Appendix D Table Measurement of the body self and S5 File: Appendix E The analyzed aspects of the body self)).

**2.4. Measurement of menopausal women’s self-stereotype**

To determine perimenopausal women’s self-stereotype, we used a survey (S6 File: Appendix F Measurement of menopausal self-stereotype) developed for the purpose of this study [41]. Next, to identify participants’ attitudes toward the menopausal woman, we adopted the following criteria based on literature review [6,10,14,15,19,22,23,42–44] and the findings of a previous pilot study [41]:

- first, we analyzed the types of associations (positive and negative) that participants presented in connection to the onset of menopause in their lives;
- second, we took account of whether participants perceived menopause as a *stage versus non-stage* in their personal development; and
- third, we identified the traits that participants attributed to the menopausal woman.

This permitted us to distinguish two types of the self-stereotype presented by menopausal women: positive self-stereotype, demonstrated by 44 women (22% of the sample), and negative self-stereotype, shown by 157 women (78% of the sample).

### Procedure

The study type was cross-sectional. The recruitment process was as follows. First, we looked for participants in schools, subsequently visiting the schools and asking the women to take part in the study. We also made an attempt to recruit nurses in local hospitals. Unfortunately, an insufficient number of only 12 women agreed to participate, although the questionnaires they filled out proved a useful resource for further analyses. Having consented to the study, the school teachers were informed about the anonymous character of the study and their being allowed to withdraw at any time.

Participation was voluntary, anonymous and individual, and the women were informed about being able to drop out at any time. The study took place between May 2011 and July 2013 in Wrocław. The study was carried out individually. Participants were instructed on how to fill out the questionnaire, which they did individually at school or at home. We held an information meeting in a school for teachers, where we described the study in detail, as well as explaining the scope of data we needed to acquire, how the data would be stored, and how we would protect the data against unauthorized access. The average questionnaire completion time was two hours. The questionnaires were filled out in any order. Participants were given a week’s time to fill out the questionnaires. Once a week, on days previously agreed on with the respondents, we visited the schools and collected the completed questionnaires. Of the 211 teachers we talked to, 210 expressed a wish to participate in the study. Eventually, we collected valid questionnaires from 201 women, as 9 of the teachers dropped out in the process. They only filled out the questionnaires on menopausal symptoms and quality of life, making their documents unsuitable for analysis on account of providing insufficient data.

### Statistical analysis

We used *Statistica 10* software and performed a structural analysis by using neural networks with structure optimization via genetic algorithms (GAS) [46]. Using the neural networks method enabled us to determine both direct and indirect predictors of participants’ SQH [46]. Neural networks is a nonlinear, advanced modelling technique capable of transforming highly complex functions. Owing to their capacity to learn by example, neural networks are able to construct the necessary models on their own. Specifically, the process of collecting representative data, which shows how a given correlation manifests, initializes a *training algorithm*, which then leads to the necessary data structure being automatically created in the network’s memory. The network relies on that data structure to utilize the newly-created model.

Importantly, using artificial neuro-fuzzy networks permits model optimization in order to turn imprecise and uncertain data into sharp and consistent output data [47].

An additional advantage of using neural networks over other statistical methods is that they allow for reducing statistical problems related to non-linearity when analyzing multiple variables (as was the case in this study). It is also worth emphasizing that when numerous variables are involved, relying on path analysis may generate erroneous and overly complicated models.

Neural networks may be used when there exists an actual relationship between dependent and independent variables, one that is non-expressible via correlations or inter-group differences. A good example of statistical analyses based on neural networks were those used to create the structure of 120 psychopathological symptoms of the twelve DSM-IV diagnostic categories. It has been concluded that complicated network models better demonstrate the complex phenomenon of psychopathological symptoms than do traditional methods of statistical analysis [48]. Another study made use of neural networks to identify the factors aggravating menopausal symptoms, concluding that factors such as low education, high body mass index and the presence of chronic disorders may all intensify the symptoms of menopause [49].

Fuzzy sets were used in the inference block and each trait was initially subject to fuzzification by use of three triangular membership functions.

New basic distribution was obtained for variables following the optimization via genetic algorithms of the shape and distribution of function of membership to fuzzy sets [50]. Apart from allowing for a soft description of uncertain and imprecise data, fuzzy sets also permit the modeling of variable distributions, which evade evaluation by more traditional statistical methods [47]. By using triple cross-validation we were able to obtain a population model that could be regarded as having two dimensions [51]. The first was comprised of the following dependent variables: global sense of qualify of life and its spheres, frequency and intensity of menopausal symptoms, as well intensity of positive and negative affect. The second, in turn, included the following independent variables: personality traits, self-esteem, menopausal woman’s self-stereotype, and strength of the body self and its aspects. We assumed that interaction existed between the variables in both dimensions.

First, we divided the set of 201 data into three sets (A (N = 67), B (N = 67), C (N = 66)). In the neuro-fuzzy network learning cycle, sets A and B were learning, and set C was testing. Then sets A and C were learning and set B was testing. Finally, sets B and C were learning and set A was testing. The learning of the regression model, and the change in learning and network parameters (including the shape and distribution of function of membership to fuzzy sets) continued until comparable models were obtained in each of the three attempts. This yielded a model that is neither overlearned nor does it overfit the data. Instead, it reflects the variable interactions occurring in the sample under examination.

## Results

The results obtained by participants on all the questionnaires had normal distribution. The questionnaire-derived raw scores were converted into sten scores, with the exception of the sense of quality of life questionnaire, in which case T-scale scores were used.

The neural networks analysis was preceded by a determination of the basic parameters of the variables analyzed. Mean age was 50.11±3.07. Of the 201 women taking part in the study, 49 were in the early and 152 in the late stage of perimenopause. The level of participants’ life satisfaction was average, as was the frequency and intensity of their menopausal symptoms. The women additionally demonstrated a low level of positive affect (27.78±6.97) and a high level of negative affect (24.83±8.18).

With regard to the analyzed personality traits: the level of neuroticism was high (107.60±27.05), of extraversion–moderate (97.26±22.32), of openness–high (106.41±21.57), of agreeableness–low (108.04±24.27), of conscientiousness–moderate (110.27 ±24.59), and of self-esteem–moderate (28.66±4.63).

With respect to the analyzed dimensions of the body-self: no or little dysfunction related to perceiving stimuli generating too weak a sensation or no sensation at all (1.46±0.52), no or little dysfunction related to perceiving stimuli generating excessively strong sensations (1.74±0.74), no dysfunction related to the interpretation of sensations in terms of physical states (2.49±0.73), difficulty in interpreting bodily sensations in terms of emotions (2.95±0.71), no or little difficulty in interpreting bodily sensations in terms of a distorted sense of physical identity (1.96±0.87), difficulty in regulation of emotions (2.95±0.71), difficulty in regulation of physical states (2.72±0.66), negative attitude toward the body (2.78±0.88), lack of comfort with closeness with others (3.10±0.53), body protection (2.18±0.55), and a moderate level of the body-self (2.39±0.49). Positive self-stereotype of the menopausal woman was displayed by 44 of the 157 women under examination (see Table 1).

**Table 1:** Descriptive statistics of the variables under study.

Characteristic	Mean ± SD
Age (years)	50.11 ± 3.07
Menopausal status (early/late)	(49/152)
**Dimensions of SQH**	
Sense of quality of life	↔ 169.38 ± 27.99
Biological sphere (psycho-physical)	↓ 37.89 ± 9.49
Social sphere (psychosocial)	↔ 43.22 ± 7.76
Subjective sphere	↔ 43.15 ± 7.36
Metaphysical sphere	↔ 45.05 ± 6.88
Frequency of psychological symptoms	↔ 23.59 ± 13.54
Frequency of vasomotor symptoms	↔ 14.71 ± 9.91
Frequency of somatic symptoms	↔ 15.32 ± 8.49
Intensity of psychological symptoms	↔ 25.80 ± 13.93
Intensity of vasomotor symptoms	↔ 15.22 ± 9.88
Intensity of somatic symptoms	↔ 16.54 ± 8.74
Intensity of positive affect	↓ 27.78 ± 6.97
Intensity of negative affect	↑ 24.83 ± 8.18
**Determinants of SQH**	
Personality traits	
Neuroticism	↑ 107.60 ± 27.05
Extraversion	↔ 97.26 ± 22.32
Openness to experience	↑ 106.41 ± 21.57
Agreeableness	↓ 108.04 ± 24.27
Conscientiousness	↔ 110.27± 24.59
Self-esteem	↔ 28.66 ± 4.63
Body self	
Raised sensation thresholds	↓ 1.46 ± 0.52
Lowered sensation thresholds	↓ 1.74 ± 0.74
Interpretation of sensations in terms of physical states	↔ 2.49 ± 0.73
Interpretation of sensations in terms of emotional states	↔ 2.49 ± 0.88
Interpretation of sensations in terms of a sense of physical Identity	↓ 1.96 ± 0.87
Regulation of emotions	↑ 2.95 ± 0.71
Regulation of physical states	↑ 2.72 ± 0.66
Attitude toward the body	↑ 2.78 ± 0.88
Comfort with physical closeness with others	↑ 3.10 ± 0.53
Body protection	↓ 2.18 ± 0.55
Strength of the Body Self	↔ 2.39 ± 0.49
Perimenopausal women’s self-stereotype (positive / negative)	(44/157)

↑ high score

↓ low score

↔ medium score

**Fig 2:**
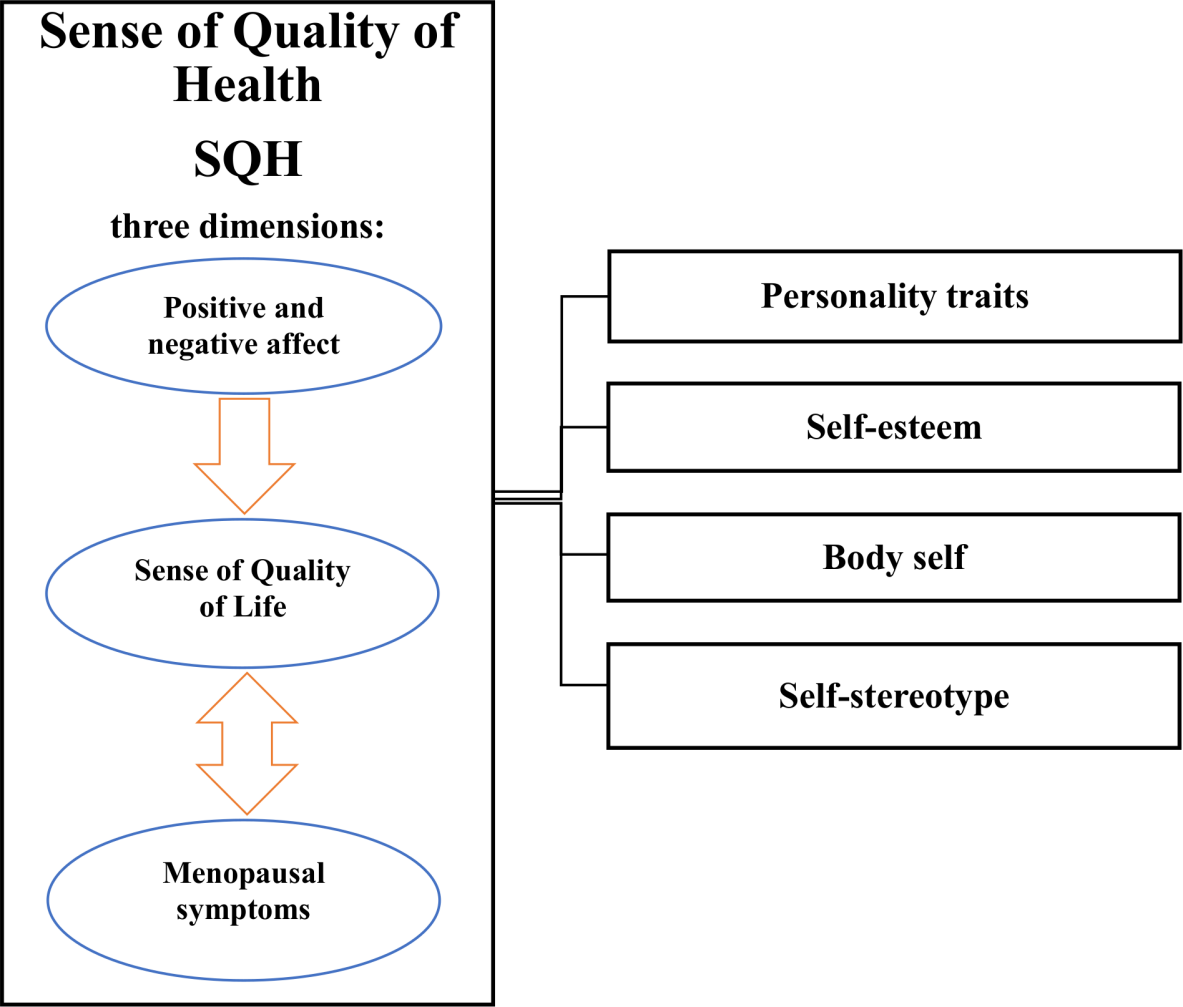
Perimenopausal women’s SQH.

We have confirmed that SQH comprises three dimensions (H1): sense of quality of life, menopausal symptoms, and positive and negative affect. In addition, structural analysis also revealed relationships between the proposed SQH dimensions. With respect to the entire model, a relationship of interaction (mutual influence) was observed between sense of quality of life, and frequency and intensity of menopausal symptoms. *β* values suggest that the higher women score on sense of quality of life and its spheres, the less frequently they experience psychological (*β* = –0.643; *Z* = –11.954; *p* = 0.01), vasomotor (*β* = –0.576; *Z* = –9.764; *p* = 0.01), and somatic (*β* = –0.544; *Z* = –7.324; *p* = 0.01) symptoms of menopause (Fig 2).

**Fig 3:**
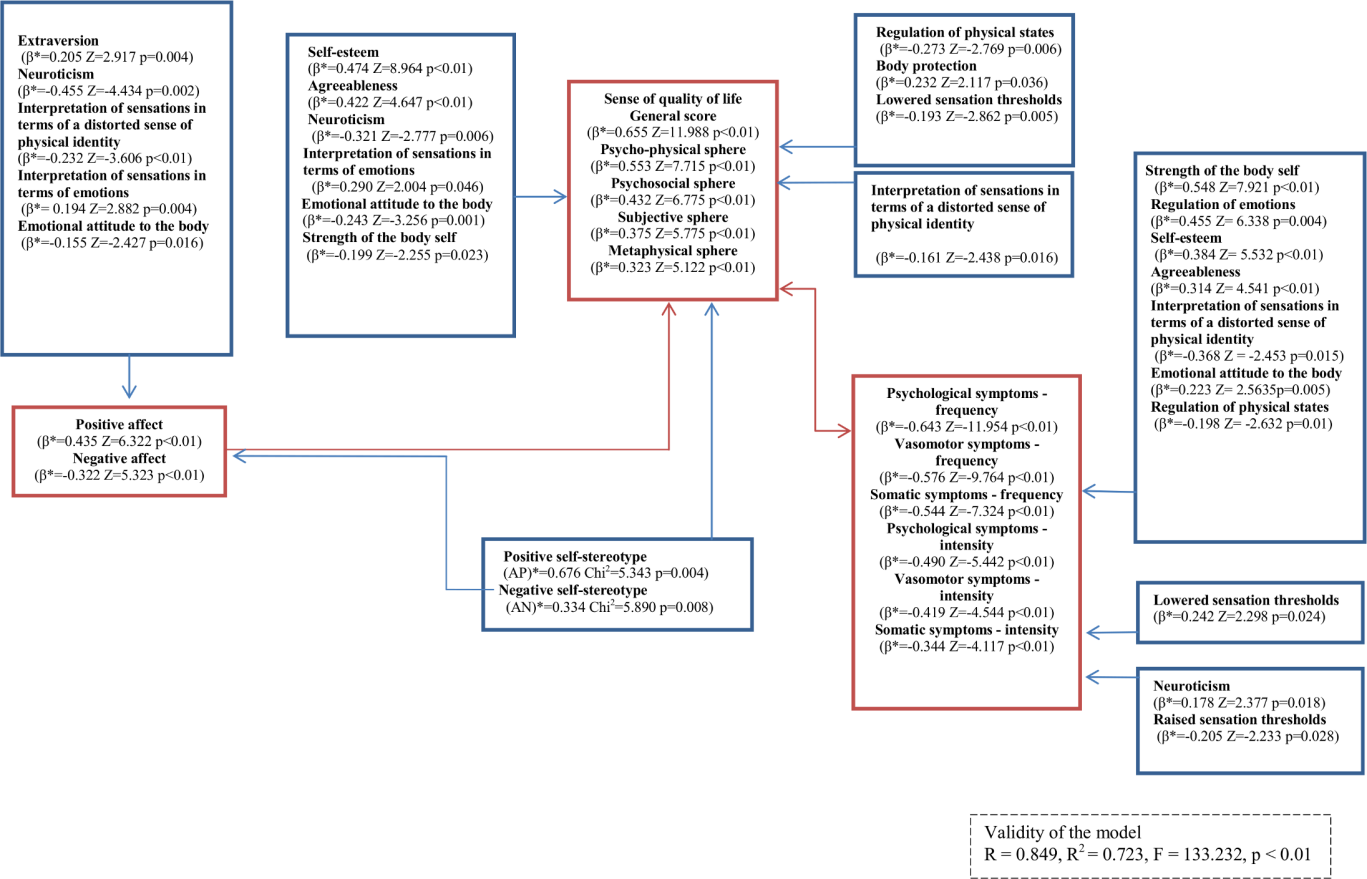
Flowchart of the multidimensional relationship using artificial neuro-fuzzy networks.

Moreover, sense of quality of life had the strongest influence on experiencing menopausal symptoms with respect to their frequency, with the influence related to their intensity being slightly weaker (see Fig 3).

Interesting to note, our analyses showed that the third SQH dimension, that is, experiencing positive and negative affect, only had an impact on the sense of quality of life (and its spheres). As it turned out, the higher the level of positive affect (*β* = 0.435; *Z* = 6.322; *p* < 0.01), the higher the level of global sense of quality of life and its spheres. At the same time, we observed a decrease in the level of negative affect (*β* = -0.322; *Z* = 5.323; *p* < 0.01). In this light, we concluded that the level of positive and negative affect was a modifier of participants’ global sense of quality of life.

The model, which was also used to verify the remaining hypotheses, was significant (R = 0.849; R^2^ = 0.723; F = 133.232; *p* < 0.01) and revealed multiple relationships existing between the variables under study, that is, causal structures, including the influence of the independent on dependent variables, which were also found to be related with each other. A detailed graph is presented in Fig 3.

In the model, variables are described by three parameters: *β*–describes the direction and strength of a given variable’s relationships within a variable group, including interaction between the variables in that group; *Z*–is a measure of a given variable’s significance for a dependent variable or a group of variables under test, including interaction between that variable and other variables; and *p*–showing the significance level for the *Z*-statistics. We also confirmed that a higher level of the sense of quality of life (along with its psycho-physical, psycho-social, subjective and metaphysical spheres) is associated with higher levels of extraversion, openness, agreeableness, conscientiousness, and self-esteem, lower levels of neuroticism and less dysfunction of the body-self functioning, and a positive self-stereotype of the menopausal woman (as suggested within H2). Detailed data about the strength and direction of revealed relations is included in Fig 3.

In the context of our third hypothesis (H3), we have shown that less frequent and less intense psychological, vasomotor and somatic symptoms of menopause are associated with higher levels of extraversion, openness, agreeableness, conscientiousness, and self-esteem, lower levels of neuroticism and less dysfunction of the body-self functioning (lower score), and a positive self-stereotype of the menopausal woman. Through its direct impact on the global sense of quality of life and its spheres, the menopausal woman’s self-stereotype is an indirect predictor of the frequency and intensity of menopausal symptoms. This is because the higher the probability of a participant showing a positive self-stereotype of the menopausal woman ((positive self-stereotype)*> 0.676 *Chi*^2^ = 5.343; *p* = 0.004) and, at the same time, the lower the probability of the participant showing a negative self-stereotype ((negative self-stereotype)*< 0.334 *Chi*^2^ = 5.890; *p* = 0.008), the greater the decrease in both frequency and intensity of psychological, vasomotor, and somatic symptoms of menopause. Fig 3 additionally shows the relationships revealed under H3.

We also demonstrated that higher positive affect is associated with higher levels of extraversion, openness, agreeableness, conscientiousness, and self-esteem, lower levels of neuroticism and less dysfunction of the body-self functioning, and a positive self-stereotype of the menopausal woman (as suggested within H4).

A the same time, higher negative affect is associated with lower levels of extraversion, openness, agreeableness, conscientiousness, and self-esteem, higher levels of neuroticism and more dysfunction of the body-self functioning, and a negative self-stereotype of the menopausal woman (as stated within H5). Additional detailed results are included in Fig 3.

## Discussion

The aim of the study was, first, to confirm that SQH comprises the three dimensions of sense of quality of life, menopausal symptoms, and positive and negative affect, and, second, to identify the predictors of SQH.

The findings of the neutral networks analysis allow for the conclusion that SQH does, indeed, comprise the three postulated dimensions.

Perimenopausal women’s SQH is largely dependent upon personality traits, self-esteem, the body self, and self-stereotype. These findings corroborated the results of previous studies, especially those emphasizing the role of internal variables for women’s health [1–26, 33,52–67].

Among predictors of SQH, we found that higher level of the sense of quality of life (along with its psycho-physical, psycho-social, subjective, and metaphysical spheres) is associated with higher levels of extraversion, openness, agreeableness, conscientiousness, and self-esteem, lower levels of neuroticism and less dysfunction of the body-self functioning (lower score), and a positive self-stereotype of the menopausal woman.

Sense of quality of life and all its dimensions (psycho-physical, psycho-social, and subjective spheres) are primarily determined by self-esteem and personality traits. This is in line with the data reported in the literature. For example, a positive correlation has been found between high self-esteem and life satisfaction [36]. With respect to personality traits, Costa and McCrae assume that extraversion and neuroticism are permanent and to a large extent inherent predispositions to directly shape life satisfaction through a perception of events that is conducive to an extraverted person perceiving the world as better, and to a neurotic person as worse than it actually is [59]. Neuroticism and extraversion could, therefore, be regarded as inherent predispositions to a higher or lower life satisfaction [59].

Perceiving one’s life in negative or positive terms is associated with two affective mechanisms that determine the predispositions to experiencing a sense of happiness [59]. These are positive emotionality (affectivity), associated with extraversion, and negative emotionality, which is close to neuroticism. The former is responsible for the level of well-being on positive dimensions, that is, for the number of positive emotional experiences as well as the general sense of well-being and the degree of satisfaction [59]. In turn, the latter determines the volume of the negative aspects of well-being, such as depression, anxiety, and the number of negative emotional experiences [59]. Neuroticism and extraversion are associated with life satisfaction not only directly but also indirectly, determining the way of perceiving (interpreting) life events as well as the way of coping with stress.

Moreover, both personality traits have a bearing on the general activity of individuals and their relations with others. Extraversion may play a dual role by serving as a buffer to the negative impact of stressors on the volume of experienced tension and by decreasing the intensity of experienced stress, thereby increasing the level of life satisfaction. In addition, extraversion is conducive to establishing and maintaining social relations, thus boosting life satisfaction, as well as facilitating good relations with others, providing the individual with a sense of happiness [59]. Conversely, neuroticism is correlated with poorer relations with others, which is a likely source of a reduced sense of well-being and low life satisfaction.

We also confirmed the third hypothesis, according to which less frequent and less intense psychological, vasomotor and somatic symptoms of menopause are associated with higher levels of extraversion, openness, agreeableness, conscientiousness, and self-esteem, lower levels of neuroticism and less dysfunction of the body-self functioning (lower score), and a positive self-stereotype of the menopausal woman.

Our findings showed that frequency and intensity of menopausal symptoms depends on the following: personality traits, including neuroticism, agreeableness and self-esteem, sense of quality of life, and women’s functioning on the particular dimensions of the body self, namely, strength of the body self, interpretation of sensations in terms of a distorted sense of physical identity, emotional attitude toward the body, regulation of emotions and physical states, as well as lowered and raised sensation thresholds.

We found that the way women experience the symptoms of menopause is related to all the parameters of the body self. A decreased intensity and frequency of the symptoms is associated with a tendency to depreciate the strength of one’s arousal and noticing few changes in the body, both of which are characteristic of persons with a strong body self. In contrast, an increased intensity and frequency of experienced symptoms can be associated with a tendency to take overt notice of the sensations coming from the body and to exaggerate the strength of arousal. Given the unpleasant bodily sensations they produce, intense and frequent symptoms of menopause may have women adopt negative attitudes toward their bodies. These, according to McKinley and Lyon, may in turn lead to experiencing negative affect and decreased well-being [14].

The importance of individual factors in perceiving and interpreting bodily sensations as well as moderating their intensity is emphasized by conceptions that describe the mechanisms linking personality and state of health [60]. For instance, Bosworth et al. have found that neurotic individuals pay increased attention to their bodies and bodily sensations, which they then interpret in terms of disorders [67]. Perceiving and interpreting bodily sensations in terms of menopausal symptoms plays an important role in how women experience the perimenopausal period in general.

This mechanism is described in great detail by the Multifactorial model as applied to the development of bodily experiences and mood states during the climacteric [15]. The model makes it necessary to understand menopausal symptoms in terms of a psychological and/or perceptual experience because “the process of perceiving and evaluating symptoms is mediated by factors such as excessive attention to internal stimuli, selective monitoring of illness-related symptoms, the context in which sensory data occur, previous experiences, social information on the relationship between complaints and illness and learned expectations” [15].

Other studies have also demonstrated that cultural context serves as a basis for women’s interpreting the changes affecting their bodies in terms of symptoms of menopause [15,62]. At the same time, however, women may treat the perceived symptoms as a source of information on the bodily changes [15]. The way they experience their own bodies is to a large extent then shaped by the fact that they compare the looks of their bodies with the culturally accepted ideal [14]. As well as determining the expectations and interpretation of physiological changes affecting the perimenopausal woman’s body, cultural factors shape women’s beliefs about what femininity and menopause are, and what they mean for them. Information about menopause feeds back to reinform them about the physical ideal of femininity [14]. Middle-aged women concentrate on their bodies, observe them and are mindful of the changes that are to come [33]. Moreover, it turns out that a lower number of experienced menopausal symptoms is associated with a greater satisfaction with one’s appearance [14]. Having negative beliefs about menopause may be conducive to focusing overly on its symptoms, which in turn is likely to modify their perceived severity [6,33].

Another hypothesis we managed to confirm was hypothesis four, which assumed that higher positive affect is associated with higher levels of extraversion, openness, agreeableness, conscientiousness, and self-esteem, lower levels of neuroticism and less dysfunction of the body-self functioning (lower score), and a positive self-stereotype of the menopausal woman.

Moreover, we also confirmed the final hypothesis, according to which higher negative affect should be associated with lower levels of extraversion, openness, agreeableness, conscientiousness, and self-esteem, higher levels of neuroticism and more dysfunction of the body-self functioning (lower score), and a negative self-stereotype of the menopausal woman.

There is plenty of research on the menopausal woman stereotypes [10,22–26,42–44], showing that attitudes to menopause, through their relationship with experienced affect, may indeed have significant implications for health promotion among menopausal women [23]. For example, Barth-Olofsson and Collins demonstrated that negative affect correlated with negative attitude to menopause, life stress and poor health, among others [68]. The role of affect for the well-being of menopausal women has received special attention in Hunter and Rendall’s cognitive behavioral model of menopausal hot flushes, under which experiencing menopausal symptoms may be dependent on several factors having a direct influence on such experiences [6]. These include mood and emotional reactions, thoughts and beliefs about menopause, experienced stress, lifestyle, as well as bodily and physiological changes. These researchers pay particular attention to the relationships between mood and self-esteem levels, and hot flushes. Lowered mood and low self-esteem imply a negative perception of menopausal symptoms, which in turn is related to increased frequency and intensity of experienced hot flushes [6].

Consistent with studies by Costa and McCrae, extraversion and neuroticism have been revealed as the strongest determinants of experiencing positive and negative affect [34,64]. Individuals demonstrating a set of personality traits that include depression, anger, hostility and fearfulness, may be characterized as having a disease-prone personality [6]. With respect to this theory, researchers have found that women who failed to demonstrate experienced fear also had poorer physical health. Similarly, women who described their lives as more stressful experienced more depression symptoms and were characterized by poorer health status. Depressive mood was correlated with life events as well as level of stress, attitudes to menopause and certain personality traits [65]. Woods and Mitchell demonstrated that onset of depression correlated with stressful life events and health status but not with hormonal changes. Moreover, women with low self-esteem were also found to experience depression symptoms and anxiety, and to have poorer physical health [34,68].

### Limitations

There are several reasons why the findings presented above must be interpreted with a degree of caution. First, the sample was confined to married women with higher education living in a large Polish city, while SQH determinants may differ for women enjoying different socio-economic status. This may likely impact on the generalizability of our findings. Second, the sample was fairly small relative to the number of variables studied. Third, the questionnaires measuring sense of quality of life and the body self have only been statistically evaluated in Poland and are yet to be used abroad. Fourth, the study made no use of variables concerning experienced stress, a factor that is often cited as impacting on the experience of menopause [23].

In practical terms, our findings may help raise awareness among women and medical practitioners, calling for a holistic approach to the health of menopausal women. Rather than limiting one’s perspective to the experienced symptoms, account should also been taken of life satisfaction, personality traits, self-esteem and experienced emotions, among other factors.

Such therapeutic action could be focused on working with the body, as the body self is suggested to be an important determinant of health [66].

The present study has also demonstrated that self-esteem plays a vital role for menopausal women’s sense of quality of life and its spheres and negative affect. Therefore, health promotion may benefit from action directed at building and strengthening self-esteem.

By undertaking a range of specific intervention techniques, which take account of the present results, for example, in cognitive-behavioral therapy (CBT), practitioners could prevent the creation of a negative self-stereotype of the menopausal woman, thus reducing the risk of severe symptoms of menopause and depression.

Further research could benefit from a thorough analysis testing the SQH model on a group of women enjoying a differential menopausal status and diverse education. For instance, it is worth checking whether intervention based on the findings presented here does indeed help women transition through menopause. Another interesting contribution could come from testing the role that sexual functioning plays on perimenopausal women’s SQH.

## Conclusions

In the present paper we have proven that SQH comprises three dimensions: sense of quality of life, menopausal symptoms, and positive and negative affect. Moreover, we showed that SQH is significantly predicted by the strength of the body self, regulation of emotions, regulation of physical states, interpretation of sensations in terms of emotions, interpretation of sensations in terms of a distorted sense of physical identity, attitudes toward the body, and raised and lowered sensation thresholds. We additionally demonstrated an interactive relationship between sense of quality of life and frequency and intensity of menopausal symptoms. Apart from the purely scientific value, our findings may have significant practical implications, as they may facilitate the creation of both prevention and therapeutic programs for women transitioning through menopause.

## Supporting information

S1 File Appendix A menopausal status survey. (DOCX)

S2 File Appendix B measurement quality of life. (DOC)

S3 File Appendix C measurement of menopausal symptoms. (DOC)

S4 File Appendix D measurement of the body self. (DOCX)

S5 File Appendix E the analyzed aspects of the body self. (DOCX)

S6 File Appendix F measurement of menopausal self-stereotype. (DOC)

S7 File Data file. (XLSX)

S8 File PANAS English version. (PDF)

S9 File Key My Measurement of menopausal symptoms. (DOCX)
