# Supplementary material for: Searching for predictors of sense of quality of health: A study using neural networks on a sample of perimenopausal women
Source: PLoS One. 2019 Jan 3;14(1):e0200129. doi: 10.1371/journal.pone.0200129 (PMC6317781; doi:10.1371/journal.pone.0200129)
Supplement: S1 File — (DOCX) [file pone.0200129.s001.docx]

**Menopausal status survey**

**You are kindly asked to answer the following questions**

**Have you been menstruating within the past 12 months?**

………………………………………………………………………………………………………

**When was the last time you menstruated?**

………………………………………………………………………………………………………**What is the reason why you stopped menstruating?**

………………………………………………………………………………………………………

**Have you been using Hormone Replacement Therapy? If so, for how long?**

……………………………………………………………………………………………………

**Why did you decide to use Hormone Replacement Therapy?**

………………………………………………………………………………………………………

**Thank you for your participation!**
