# Supplementary material for: Searching for predictors of sense of quality of health: A study using neural networks on a sample of perimenopausal women
Source: PLoS One. 2019 Jan 3;14(1):e0200129. doi: 10.1371/journal.pone.0200129 (PMC6317781; doi:10.1371/journal.pone.0200129)
Supplement: S2 File — (DOC) [file pone.0200129.s002.doc]

*Maria Straś – Romanowska, Anna Oleszkowicz, Tomasz Frąckowiak***KWESTIONARIUSZ POCZUCIA JAKOŚCI ŻYCIA**

**Sense of quality of life questionnaire**

First and last name ( or personal code ) ………………………………....... Sex: Male Female

Today’s Date: ………………………. Date of birth: …………………………..

This is an assessment referring to different aspects of a human being (see below). There are four possible answers next to each question. Please read the questions carefully and circle the answer which best describes your situation.
For each sentence, please choose only one answer as follows.

| 1 | Physical disorders disturb me in my daily life | 1 2 3 4 |
| --- | --- | --- |
| 2 | There are some people with who I am deeply connected to | 1 2 3 4 |
| 3 | I have no interest or hobby on which I spend a lot of time | 1 2 3 4 |
| 4 | I agree that not everything depends on me | 1 2 3 4 |
| 5 | I’m usually energetic and full of verve | 1 2 3 4 |
| 6 | In my life there are more triumphs than defeats | 1 2 3 4 |
| 7 | When I am making an important choice, I consider what other people say first | 1 2 3 4 |
| 8 | There are some things that fascinate me with their beauty | 1 2 3 4 |
| 9 | I sleep well | 1 2 3 4 |
| 10 | I fit in with people who are around me | 1 2 3 4 |
| 11 | I have a sense that I found my place in life | 1 2 3 4 |
| 12 | I sympathize with miserable, poor, and unhappy people | 1 2 3 4 |
| 13 | I slowly mobilize myself to an effort and lose plenty of time before I start working | 1 2 3 4 |
| 14 | In controversial situations I rarely decide to compromise | 1 2 3 4 |
| 15 | Even small things make me happy | 1 2 3 4 |

1 – completely disagree

2 – somewhat disagree

3 – somewhat agree

4 – completely agree

| 16 | I believe that I am guided by some form of higher power | 1 2 3 4 |
| --- | --- | --- |
| 17 | In my free time I can relax easily and know how to rest | 1 2 3 4 |
| 18 | I am important to others | 1 2 3 4 |
| 19 | I like what I do | 1 2 3 4 |
| 20 | In my life nothing happens by chance | 1 2 3 4 |
| 21 | I am in good physical condition | 1 2 3 4 |
| 22 | My family life is satisfying | 1 2 3 4 |
| 23 | I am responsible for what I do | 1 2 3 4 |
| 24 | I have values which help me remain hopeful, even in difficult moments | 1 2 3 4 |
| 25 | I rarely get sick | 1 2 3 4 |
| 26 | People respect what I do | 1 2 3 4 |
| 27 | Have to need to do something important in my life | 1 2 3 4 |
| 28 | I wish to leave something good behind after I’m gone | 1 2 3 4 |
| 29 | I usually feel tired, exhausted and with no energy | 1 2 3 4 |
| 30 | I often meet people who are friendly towards me | 1 2 3 4 |
| 31 | My life depends mainly on me | 1 2 3 4 |
| 32 | In spite of there being plenty of evil in the world, there is a lot of good as well | 1 2 3 4 |
| 33 | I am often in the hospital | 1 2 3 4 |
| 34 | I mean a lot to the people who are around me | 1 2 3 4 |
| 35 | I don’t have established plans for the future | 1 2 3 4 |
| 36 | I rarely give up personal pleasure in favor of more important values | 1 2 3 4 |
| 37 | I often suffer from pain | 1 2 3 4 |
| 38 | In the event of trouble, I can depend only on myself | 1 2 3 4 |
| 39 | Gaining new experiences gives me a lot of joy | 1 2 3 4 |
| 40 | It is important to work to better oneself | 1 2 3 4 |
| 41 | I eat well | 1 2 3 4 |
| 42 | I feel lonely | 1 2 3 4 |
| 43 | I have a clear aim in my life, and I am determined to achieve it | 1 2 3 4 |

1 – completely disagree

2 – somewhat disagree

3 – somewhat agree

4 – completely agree

| 44 | It is difficult for me to forgive others | 1 2 3 4 |
| --- | --- | --- |
| 45 | I’m happy with my physical appearance | 1 2 3 4 |
| 46 | I feel necessary to other people | 1 2 3 4 |
| 47 | I almost always say what I think | 1 2 3 4 |
| 48 | I believe that my life doesn’t finish with my death | 1 2 3 4 |
| 49 | I am in good health | 1 2 3 4 |
| 50 | I often have conflicts with other people | 1 2 3 4 |
| 51 | In my life I follow the path I have chosen for myself | 1 2 3 4 |
| 52 | In spite of adversity, I think that my life has a deep meaning | 1 2 3 4 |
| 53 | My illness doesn’t allow me to look calmly towards the future | 1 2 3 4 |
| 54 | In my contacts with other people, there is often fighting and competition | 1 2 3 4 |
| 55 | I accept myself as I am | 1 2 3 4 |
| 56 | A desire for personal reflection helps me in life | 1 2 3 4 |
| 57 | I am physically active (play sports, gymnastics, go for walks) | 1 2 3 4 |
| 58 | I feel connected with person intimate to me | 1 2 3 4 |
| 59 | I often do something which I later regret | 1 2 3 4 |
| 60 | My life is valuable, even if not all goes as planned | 1 2 3 4 |

1 – completely disagree

2 – somewhat disagree

3 – somewhat agree

4 – completely agree
