# Supplementary material for: Searching for predictors of sense of quality of health: A study using neural networks on a sample of perimenopausal women
Source: PLoS One. 2019 Jan 3;14(1):e0200129. doi: 10.1371/journal.pone.0200129 (PMC6317781; doi:10.1371/journal.pone.0200129)
Supplement: S3 File — (DOC) [file pone.0200129.s003.doc]

**Janette M. Perz**

**MY SENSE OF WELL-BEING**

**Adapted by Eleonora Bielawska-Batorowicz**

Below are 25 statements about well-being. Mark an X in the box representing **how often** you have behaved or felt in a given way in the past 3 months. Use the following scale:

**Frequency rating**

0-NEVER (not even once in the past 3 months)

1-RARELY (once or twice in the past 3 months)

2-SOMETIMES (about 5 times in the past 3 months)

3-OFTEN (between 5 and 10 times in the past 3 months)

4-VERY OFTEN (more often than 10 times in the past 3 months)

5-ALMOST ALWAYS (almost every day in the past 3 months)

If you have behaved or felt in the way described by below statements, please mark an X in the box representing **how intense** the given sensation was. Use the following scale:

**Intensity rating**

0-DOES NOT APPLY (the sensation did not occur)

1-SLIGHT (a barely noticeable change or sensation)

2-LOW (a small change, a weak sensation)

3-MODERATE (a noticeable change / sensation)

4-HIGH (a clearly noticeable change and an intense sensation)

5-VERY HIGH (a radical change and an extremely intense sensation)

Please do not take too long and do not skip any statement.

|  | **FREQUENCY** | | | | | |  | **INTENSITY** | | | | | |
| --- | --- | --- | --- | --- | --- | --- | --- | --- | --- | --- | --- | --- | --- |
| **SENSATION** | **0** | **1** | **2** | **3** | **4** | **5** |  | **0** | **1** | **2** | **3** | **4** | **5** |
| 1. Worrying for no reason  (feeling concerned and upset without reason) |  |  |  |  |  |  |  |  |  |  |  |  |  |
| 2. Depressed feelings (feeling unhappy and sad without reason) |  |  |  |  |  |  |  |  |  |  |  |  |  |
| 3. Tightness or pressure in the head or other body parts  (feeling pressure in the head or whole body) |  |  |  |  |  |  |  |  |  |  |  |  |  |
| 4. Sleeplessness (difficulty in falling asleep, interrupted sleep) |  |  |  |  |  |  |  |  |  |  |  |  |  |
| 5. No feeling in hands and feet (numbness or lack of sensation in hands and feet) |  |  |  |  |  |  |  |  |  |  |  |  |  |
|  |  |  |  |  |  |  |  |  |  |  |  |  |  |
|  | **FREQUENCY** | | | | | |  | **INTENSITY** | | | | | |
| **SENSATION** | **0** | **1** | **2** | **3** | **4** | **5** |  | **0** | **1** | **2** | **3** | **4** | **5** |
| 6. Loss of sexual interest  (loss of interest in sex and sexual desire) |  |  |  |  |  |  |  |  |  |  |  |  |  |
| 7. Poor concentration  (trouble concentrating on the task at hand) |  |  |  |  |  |  |  |  |  |  |  |  |  |
| 8. Palpitations  (quick or strong heartbeat) |  |  |  |  |  |  |  |  |  |  |  |  |  |
| 9. Cold hands and feet  (having cold hands and feet even when the rest of the body is warm) |  |  |  |  |  |  |  |  |  |  |  |  |  |
| 10. Moodiness  (changes in mood and emotional states for no reason) |  |  |  |  |  |  |  |  |  |  |  |  |  |
| 11. Excitability (becoming easily aroused or nervous) |  |  |  |  |  |  |  |  |  |  |  |  |  |
| 12. Poor appetite  (loss of interest in and aversion to food) |  |  |  |  |  |  |  |  |  |  |  |  |  |
| 13. Constipation  (difficulty in emptying the bowels) |  |  |  |  |  |  |  |  |  |  |  |  |  |
| 14. Weight gain  (5% weight increase in the past year) |  |  |  |  |  |  |  |  |  |  |  |  |  |
| 15. Irritability  (becoming upset or annoyed easily) |  |  |  |  |  |  |  |  |  |  |  |  |  |
| 16. Waking up early in the morning  (waking up early and trouble in falling asleep again) |  |  |  |  |  |  |  |  |  |  |  |  |  |
| 17. Shortness of breath  (difficulty breathing, feelings of running out of breath) |  |  |  |  |  |  |  |  |  |  |  |  |  |
| 18. Numbness and tingling  (loss of sensation or numbness and prickling in any part of the body) |  |  |  |  |  |  |  |  |  |  |  |  |  |
| 19. Hot flashes  (a feeling of heat that occurs suddenly and involves the chest, neck and face) |  |  |  |  |  |  |  |  |  |  |  |  |  |
| 20. Headaches  (a headache less intense than a migraine) |  |  |  |  |  |  |  |  |  |  |  |  |  |
| 21. Tense feelings  (feeling tense and anxious) |  |  |  |  |  |  |  |  |  |  |  |  |  |
| 22. Crying spells  (breaking into tears or wanting to cry with no apparent reason) |  |  |  |  |  |  |  |  |  |  |  |  |  |
| 23. Dry eyes  (feelings of dryness in the eyes) |  |  |  |  |  |  |  |  |  |  |  |  |  |
| 24. Dyspareunia  (painful sexual intercourse) |  |  |  |  |  |  |  |  |  |  |  |  |  |
| 25. Sweating  (sweating in situations other than intense physical exercise) |  |  |  |  |  |  |  |  |  |  |  |  |  |
