# Supplementary material for: Searching for predictors of sense of quality of health: A study using neural networks on a sample of perimenopausal women
Source: PLoS One. 2019 Jan 3;14(1):e0200129. doi: 10.1371/journal.pone.0200129 (PMC6317781; doi:10.1371/journal.pone.0200129)
Supplement: S4 File — (DOCX) [file pone.0200129.s004.docx]

**Part I**

Below are questions concerning different aspects of experiencing one’s body. Please rate how often the situations or experiences described happen to you using the following scale:

**1 – Never**

**2 – Very rarely (once or twice in a lifetime)**

**3 – Sometimes**

**4 – Quite often**

**5 – Very often**

**NOTE:** We are interested in experiences that do not result from somatic illness, anesthesia or taking medication or drugs. In selecting how often you have had a given experience, do not take experiences due to illness, anesthesia, etc. into account.

| 1. There are times when my whole body or part of my body is immune to pain. | 1 2 3 4 5 |
| --- | --- |
| 1. There are times when I see people and/or objects out of focus and blurred (like through a fog) or my sight is restricted (as if I were looking through a narrow tunnel). | 1 2 3 4 5 |
| 1. There are times when impressions coming from my body are dulled, coming to me as if through a fog. | 1 2 3 4 5 |
| 1. There are times when I hurt myself (get a cut, walk into something, etc.) without feeling anything. | 1 2 3 4 5 |
| 1. There are times when, although I am focused, sounds coming from close by sound as if they are coming from far away. | 1 2 3 4 5 |
| 1. There are times when I feel that my body is not “filled” with any sense impressions. | 1 2 3 4 5 |
| 1. There are times when a temperature that others consider normal causes me to feel pain. | 1 2 3 4 5 |
| 1. There are times when, being completely healthy, I stop being sensitive to smells. | 1 2 3 4 5 |
| 1. There are times when I find it hard to gauge the temperature of water (for instance when taking a bath or swimming) based on what I feel. | 1 2 3 4 5 |
| 1. There are times when objects and/or people appear extraordinarily “in focus” (contours, colors or the contrast between colors appearing with more clarity than usual). | 1 2 3 4 5 |
| 1. There are periods when wearing clothes made of soft fabric causes me to feel irritation, itching or pain. | 1 2 3 4 5 |
| 1. There are periods when my sense of smell is sharpened and smells that didn’t bother me before become irritating. (**NOTE:** aside from during pregnancy or menstruation) | 1 2 3 4 5 |
| 1. There are times when I feel like my body has become desensitized. | 1 2 3 4 5 |
| 1. There are periods when my sense of taste is sharpened and products that I enjoyed before seem too sweet, too salty or too spicy. | 1 2 3 4 5 |
| 1. There are times when even a soft touch feels painful. | 1 2 3 4 5 |
| 1. There are times when even very soft sounds (the sound of a door being lightly closed) are irritating. | 1 2 3 4 5 |
| 1. At times different types of dishes or beverages taste exactly the same. | 1 2 3 4 5 |

**Part II**

Please rate each statement on the following scale:

**1 – I definitely disagree**

**2 – I somewhat disagree**

**3 – I’m not sure**

**4 – I somewhat agree**

**5 – I definitely agree**

If possible, please avoid picking answer 3 and use it only if no other answer is applicable.

| 1. It’s hard for me to say whether the fact that I don’t feel as well as I usually do is due to illness or to some other state. | 1 2 3 4 5 |
| --- | --- |
| 1. I regularly go for medical check-ups. | 1 2 3 4 5 |
| 1. The touch of another person makes me anxious. | 1 2 3 4 5 |
| 1. There are periods when I’m unable to feel or experience anything even though I talk to people and perform various tasks.* | 1 2 3 4 5 |
| 1. There are times when I resent the way my body is functioning. | 1 2 3 4 5 |
| 1. I have the impression that my body has been taken over by some kind of an “evil and incomprehensible” force.* | 1 2 3 4 5 |
| 1. The sight of some parts of my body fills me with negative feelings like anger, shame, disgust, etc. | 1 2 3 4 5 |
| 1. Whenever I notice the first signs of illness I do something that makes me feel better. | 1 2 3 4 5 |
| 1. I don’t like spending time in places where there are a lot of people and where someone might stand close to me or touch me. | 1 2 3 4 5 |
| 1. There are times when I feel that the impressions arising in my body are not entirely mine.* | 1 2 3 4 5 |
| 1. I feel helpless against the sexual arousal I am experiencing. | 1 2 3 4 5 |
| 1. I rarely follow doctors’ recommendations precisely. | 1 2 3 4 5 |
| 1. Sometimes I hate the way I look. | 1 2 3 4 5 |
| 1. Sometimes I find myself eating in an uncontrolled manner, even though I realize I am no longer hungry. | 1 2 3 4 5 |
| 1. Sometimes I have doubts regarding the limits of my body.* | 1 2 3 4 5 |
| 1. Sometimes I feel as if I were dead inside*. | 1 2 3 4 5 |
| 1. I like my appearance, although I know it isn’t perfect. | 1 2 3 4 5 |
| 1. I feel calm and relaxed when someone I am close to is stroking my face, hands, or hair. | 1 2 3 4 5 |
| 1. I don’t understand people who are able to have fun in crowded places (e.g. clubs, concert halls). | 1 2 3 4 5 |
| 1. I would gladly change the way my body functions. | 1 2 3 4 5 |
| 1. I try to go to sleep before midnight if I have to get up early in the morning. | 1 2 3 4 5 |
| 1. There are times when I’m unable to tell whether I am full or not after a meal. | 1 2 3 4 5 |
| 1. Sometimes I get the impression that my body is “speaking a language of its own” that I am unable to understand.* | 1 2 3 4 5 |
| 1. I like physical contact with other people. | 1 2 3 4 5 |
| 1. When I am sexually aroused, I know exactly what made me feel that way. | 1 2 3 4 5 |
| 1. I carefully look in both directions when crossing the street. | 1 2 3 4 5 |
| 1. There are times when I don’t know what to respond when asked about how I feel. | 1 2 3 4 5 |
| 1. I try to keep my body weight in check. | 1 2 3 4 5 |
| 1. I usually contact a doctor when I am certain I am starting to get sick. | 1 2 3 4 5 |
| 1. Sometimes I feel that the contours of my body are starting to get blurred, as if I were losing my “shape”, i.e. my own limits.* | 1 2 3 4 5 |
| 1. I find it hard to define my feelings based on the impressions arising in my body. | 1 2 3 4 5 |

* Does not apply to experiences being the consequence of somatic illness, taking medication or drugs.

| 1. There are times when I do not notice that I am beginning to get sick. | 1 2 3 4 5 |
| --- | --- |
| 1. I have periods of sadness and dejection that start unexpectedly. | 1 2 3 4 5 |
| 1. I feel ill at ease when people are physically too close to me. | 1 2 3 4 5 |
| 1. There are body parts that I am completely unable to accept. | 1 2 3 4 5 |
| 1. There are times when I cannot define the emotion I am feeling. | 1 2 3 4 5 |
| 1. There are times, when, although I am very tired, I am unable to relax and I stay “hyped” for a very long time. | 1 2 3 4 5 |
| 1. I am inconvenienced by the functioning of my body in many situations. | 1 2 3 4 5 |
| 1. There are times when I am so anxious or angry that I am unable to concentrate on the task at hand. | 1 2 3 4 5 |
| 1. When I am joyful or happy, I know how to prolong this pleasant state. | 1 2 3 4 5 |
| 1. I try to keep my distance (physically) when talking to someone I don’t know. | 1 2 3 4 5 |
| 1. There are times when I notice that I am tired only once I am completely exhausted. | 1 2 3 4 5 |
| 1. I can usually correctly point out the cause of my physical ailments. | 1 2 3 4 5 |
| 1. There are times when, for unknown reasons, I am suddenly very hungry, although I should theoretically feel full. | 1 2 3 4 5 |
| 1. I experience feelings that I cannot at all name. | 1 2 3 4 5 |
| 1. I am ashamed of my appearance. | 1 2 3 4 5 |
| 1. There are times when I cannot tell whether changes in my body are evidence of sexual arousal. | 1 2 3 4 5 |
| 1. When I am angry at someone, I’m not really sure how or why it came about. | 1 2 3 4 5 |
| 1. There are times when I am irritated or distraught, and only upon closer thought do I realize that it’s because of not having enough sleep. | 1 2 3 4 5 |
| 1. When I am tired after a whole day of work I am able to organize my afternoon/evening in a way that allows me to relax. | 1 2 3 4 5 |
| 1. I am sometimes terrified by strange bodily sensations that I am experiencing.* | 1 2 3 4 5 |
| 1. When I am feeling strong emotions, I am unable to fall asleep even if I’m very tired. | 1 2 3 4 5 |
| 1. For unknown reasons, I am sometimes overcome by sleepiness which I am unable to control. | 1 2 3 4 5 |
| 1. There are times when I feel fear or panic, although I am unable to determine the reason for it. | 1 2 3 4 5 |
| 1. There are times when I feel as though there is nothing inside me, as if I were hollow inside.* | 1 2 3 4 5 |
| 1. There are times when I am very excited, although I don’t know what feelings this is related to. | 1 2 3 4 5 |
| 1. I often feel as though other people are coming up too close to me. | 1 2 3 4 5 |
| 1. When I feel nervous, I usually know why. | 1 2 3 4 5 |
| 1. When I am sad or dejected I find it difficult to stop feeling that way. | 1 2 3 4 5 |
| 1. When someone touches me, I feel as though they are entering my private world. | 1 2 3 4 5 |
| 1. When I feel gloomy, I usually don’t know what is amiss. | 1 2 3 4 5 |
| 1. When talking to my partner, I try to be physically close to him/her. | 1 2 3 4 5 |
| 1. I would gladly change the way I look. | 1 2 3 4 5 |
| 1. I like hugging/snuggling up to people I am close to. | 1 2 3 4 5 |
| 1. I have a tendency to keep physical distance between myself and people I am talking to. | 1 2 3 4 5 |
| 1. I can go without sleep for a few nights in a row without feeling tired. | 1 2 3 4 5 |
| 1. I find it difficult to name my emotions. | 1 2 3 4 5 |
| 1. I find it much harder to concentrate on a problem when surrounded by other people (e.g. in a library reading room). | 1 2 3 4 5 |
| 1. There are times when I feel very tired, although I cannot tell why. | 1 2 3 4 5 |

-----------------------------------------------------------------------------------------------------------------------------------------------------* Does not apply to experiences being the consequence of somatic illness, taking medication or drugs.

| 1. When I am sick, I know what to do to make myself feel better. | 1 2 3 4 5 |
| --- | --- |
| 1. There are times when I am paralyzed by fear that I cannot control. | 1 2 3 4 5 |
| 1. When I get a cut, I put on a dressing right away. | 1 2 3 4 5 |
| 1. I regularly use body care products. | 1 2 3 4 5 |
