# Supplementary material for: Searching for predictors of sense of quality of health: A study using neural networks on a sample of perimenopausal women
Source: PLoS One. 2019 Jan 3;14(1):e0200129. doi: 10.1371/journal.pone.0200129 (PMC6317781; doi:10.1371/journal.pone.0200129)
Supplement: S5 File — (DOCX) [file pone.0200129.s005.docx]

**Appendix D**

**Aspects of the body self analyzed in the study**

| Aspect of the body self | Expression of body self functioning |
| --- | --- |
| Body image | attitude towards the body, body image, emotional attitude to how the body looks and functions |
| Comfort with closeness with others | experienced level of safety and comfort with closeness with other people, both close ones and strangers |
| Body protection | refers to behaviors aimed at protecting and improving one’s health; also includes willingness to take proper care of the body |
| STRENGTH OF THE BODY SELF  capacity to perceive stimuli and interpret sensations in terms of physical states and emotions, sense of physical identity, and regulation of physical states and emotions | raised sensation thresholds, which can be defined as a feeling that a strong stimulus fails to elicit a given sensation, or that the sensation is too weak |
|  | lowered sensation thresholds, that is, a feeling that a weak stimulus elicits a sensation that is too strong, while low-intensity stimuli are perceived as irritating or painful |
|  | interpretation of sensations in terms of physical states, such as fatigue, hunger, and sleepiness; a dysfunction may be expressed through inability to identify experienced arousal as hunger or fatigue |
|  | interpretation of sensations in terms of emotions; a dysfunction may be expressed through inability to describe experienced arousal in emotional terms |
|  | interpretation of sensations in terms of a sense of physical identity: interpretation of sensations in terms of a distorted sense of physical identity, which leads to describing one’s experiences through a sensation of blurring body boundaries and emptiness, and feeling inconsistent with the body |
|  | regulation of physical states: ability to cope with bodily states (ability to satisfy these states) and knowing what causes them; a dysfunction may be expressed through feelings of strong fatigue without being aware of its cause, or feelings of sadness without knowing what has triggered them |
|  | regulation of emotions: ability to cope with emotions (ability to contain negative emotions, and elicitng and maintaining positive ones) and knowing what causes them; a dysfunction may be expressed through helplessness about one’s emotions (such as anger) or bodily states (such as sexual arousal), self-harm, or abusing alcohol to reduce negative emotions |
|  |  |

Source: own elaboration based on Sakson-Obada [41].
