# Supplementary material for: Searching for predictors of sense of quality of health: A study using neural networks on a sample of perimenopausal women
Source: PLoS One. 2019 Jan 3;14(1):e0200129. doi: 10.1371/journal.pone.0200129 (PMC6317781; doi:10.1371/journal.pone.0200129)
Supplement: S6 File — (DOC) [file pone.0200129.s006.doc]

**SURVEY**

**Dear Madam!**

**Jestem studentką III roku Studiów Doktoranckich Psychologii Uniwersytetu Wrocławskiego. Piszę pracę poświęconą psychologicznym aspektom okresu menopauzy. Niniejsza ankieta ma charakter anonimowy a wyniki uzyskane za jej pośrednictwem zostaną wykorzystane tylko i wyłącznie w celach naukowych.**

**Proszę o szczere i rzetelne odpowiedzi. Wybraną przez siebie odpowiedź proszę zakreślić lub wziąć w kółko.**

**AGE:………..years old**

**EDUCATION:**

1. primary
2. vocational
3. secondary
4. college/university degree

**CIVIL STATUS:**

1. single
2. married
3. divorced
4. widowed
5. **What associations do you have with the word ‘menopause’?**

………………………………………………………………………………………………………………………………………………………………………………………………………………………………………………………………………………………………………………………………………………………………………………………………………………………………………………………………………………………………………………………………………………………………………………………………………………………………………………………………………………………………………………………………………………………………………………………………………………………………………………………………………………………………………………………………………………………………………………………………………………………………………………………………………………………………………………………………………………………………………………………………………………………………………………

1. **Do you consider menopause to be a new stage in life? What is characteristic of this period? What is important and new in menopause as compared with the period that precedes it?**

………………………………………………………………………………………………………………………………………………………………………………………………………………………………………………………………………………………………………………………………………………………………………………………………………………………………………………………………………………………………………………………………………………………………………………………………………………………………………………………………………………………………………………………………………………………………………………………………………………………………………………………………………………………………………………………………………………………………………………………………………………………………………………………………………………………………………………………………………………………………………………………………………………………………………………

1. **How do you define your feminininty?**

………………………………………………………………………………………………………………………………………………………………………………………………………………………………………………………………………………………………………………………………………………………………………………………………………………………………………………………………………………………………………………………………………………………………………………………………………………………………………………………………………………………………………………………………………………………………………………………………………………………………………………………………………………………………………………………………………………………………………………………………………………………………………………………………………………………………………………………………………………………………………………………………………………………………………………

1. **In your opinion, does stopping to menstruate change anything in a woman’s sense of femininity?**

………………………………………………………………………………………………………………………………………………………………………………………………………………………………………………………………………………………………………………………………………………………………………………………………………………………………………………………………………………………………………………………………………………………………………………………………………………………………………………………………………………………………………………………………………………………………………………………………………………………………………………………………………………………………………………………………………………………………………………………………………………………………………………………………………………………………………………………………………………………………………………………………………………………………………………

1. **What do you think is characteristic of a menopausal woman?**

………………………………………………………………………………………………………………………………………………………………………………………………………………………………………………………………………………………………………………………………………………………………………………………………………………………………………………………………………………………………………………………………………………………………………………………………………………………………………………………………………………………………………………………………………………………………………………………………………………………………………………………………………………………………………………………………………………………………………………………………………………………………………………………………………………………………………………………………………………………………………………………………………………………………………………

1. **What positive associations do you have with a woman going through menopause?**

**………………………………………………………………………………………………………………………………………………………………………………………………………………………………………………………………………………………………………………………………………………………………………………………………………………………………………………………………………………………………………………………………………………………………………………………………………………………………………………………………………………………………………………………………………………………………………………………………………………………………………………………………………………………………………………………………………………………………………………………………………………………………………………………………………………………………………………………………………………………………………………………………………………………………………………**

1. **What negative associations do you have with a woman going through menopause?**

**………………………………………………………………………………………………………………………………………………………………………………………………………………………………………………………………………………………………………………………………………………………………………………………………………………………………………………………………………………………………………………………………………………………………………………………………………………………………………………………………………………………………………………………………………………………………………………………………………………………………………………………………………………………………………………………………………………………………………………………………………………………………………………………………………………………………………………………………………………………………………………………………………………………………………………**

**Thank you very much for completing the survey!**
