# Supplementary material for: Searching for predictors of sense of quality of health: A study using neural networks on a sample of perimenopausal women
Source: PLoS One. 2019 Jan 3;14(1):e0200129. doi: 10.1371/journal.pone.0200129 (PMC6317781; doi:10.1371/journal.pone.0200129)
Supplement: S8 File — (PDF) [file pone.0200129.s008.pdf]

Worksheet 3.1 The Positive and Negative Affect Schedule (PANAS; Watson et al., 1988)

PANAS Questionnaire

This scale consists of a number of words that describe different feelings and emotions. Read each item and then list the number from the scale below next to each word. **Indicate to what extent you feel this way right now, that is, at the present moment *OR* indicate the extent you have felt this way over the past week (circle the instructions you followed when taking this measure)**

1	2	3	4	5
Very Slightly or Not at All	A Little	Moderately	Quite a Bit	Extremely

__________	1. Interested	__________	11. Irritable
__________	2. Distressed	__________	12. Alert
__________	3. Excited	__________	13. Ashamed
__________	4. Upset	__________	14. Inspired
__________	5. Strong	__________	15. Nervous
__________	6. Guilty	__________	16. Determined
__________	7. Scared	__________	17. Attentive
__________	8. Hostile	__________	18. Jittery
__________	9. Enthusiastic	__________	19. Active
__________	10. Proud	__________	20. Afraid

Scoring Instructions:

Positive Affect Score: Add the scores on items 1, 3, 5, 9, 10, 12, 14, 16, 17, and 19. Scores can range from 10 – 50, with higher scores representing higher levels of positive affect. Mean Scores: Momentary = 29.7 (*SD* = 7.9); Weekly = 33.3 (*SD = 7.2)*

Negative Affect Score: Add the scores on items 2, 4, 6, 7, 8, 11, 13, 15, 18, and 20. Scores can range from 10 – 50, with lower scores representing lower levels of negative affect. Mean Score: Momentary = 14.8 (*SD* = 5.4); Weekly = 17.4 (*SD* = 6.2)

Copyright © 1988 by the American Psychological Association. Reproduced with permission. The official citation that should be used in referencing this material is Watson, D., Clark, L. A., & Tellegan, A. (1988). Development and validation of brief measures of positive and negative affect: The PANAS scales. Journal of Personality and Social Psychology, 54(6), 1063–1070.
