# Supplementary material for: Searching for predictors of sense of quality of health: A study using neural networks on a sample of perimenopausal women
Source: PLoS One. 2019 Jan 3;14(1):e0200129. doi: 10.1371/journal.pone.0200129 (PMC6317781; doi:10.1371/journal.pone.0200129)
Supplement: S9 File — (DOCX) [file pone.0200129.s009.docx]

**S5 Key My Measurement of menopausal symptoms**

**Janette M. Perz – My Sense of Well-Being Questionnaire**

**KEY**

| **Menopausal symptoms** | **Questions** |
| --- | --- |
| Frequency and intensity of psychological symptoms | 1,2,3,7,10,11,15,21,22, |
| Frequency and intensity of vasomotor symptoms | 5,8,9,12,17,18,19,23,25 |
| Frequency and intensity of somatic symptoms | 4,6,13,14,16,20,24 |
| Items multiplied x2 for frequency and intensity; other multiplied x1 | 2,4,5,11,14,15,18,21,24 |

**Score calculation principle**

Women rate each symptom on a 6-point scale, taking account of their frequency and intensity in the past 3 months. When rating symptom frequency, participants use the following scale: *Never* (0) – not even once in the past 3 months; *Rarely* (1) – once or twice in the past 3 months; *Sometimes* (2) – about 5 times; *Often* (3) – between 5 and 10 times; *Very often* (4) – more often than 10 times in the past 3 months; and *Almost always* (5) – almost every day in the past 3 months.

In turn, the following 6-point scale is used to rate symptom intensity: (0) – the sensation did not occur; *Slight* (1) – a barely noticeable change or sensation; *Low* (2) – a small change, a weak sensation; *Moderate* (3) – a noticeable change / sensation; *High* (4) – a clearly noticeable change and an intense sensation; *Very high* (0) – a radical change and an extremely intense sensation.

The score for each symptom is calculated by multiplying the number of points assigned to it by the assigned weight of 1 or 2.

**Temporary norms of the My Sense of Well-Being Questionnaire**

| **STEN** | **Frequency of symptoms** | | | **Intensity of symptoms** | | |
| --- | --- | --- | --- | --- | --- | --- |
|  | **psychological** | **vasomotor** | **somatic** | **psychological** | **vasomotor** | **somatic** |
| **1 very low** | **0-2** |  |  | **0-2** |  | **1** |
| **2 very low** | **3-7** | **1** | **0-2** | **3-8** | **1** | **2-3** |
| **3 low** | **8-10** | **2-3** | **3-5** | **9-11** | **2-3** | **4-6** |
| **4 low** | **11-17** | **4-7** | **6-9** | **12-16** | **4-7** | **7-10** |
| **5 average** | **18-23** | **8-13** | **10-14** | **17-24** | **8-14** | **11-14** |
| **6 average** | **24-32** | **14-19** | **15-18** | **25-33** | **15-19** | **15-18** |
| **7 high** | **33-40** | **20-25** | **19-23** | **34-40** | **20-25** | **19-24** |
| **8 wysoki** | **41-47** | **26-33** | **24-28** | **41-46** | **26-32** | **25-29** |
| **9 very high** | **48-52** | **34-38** | **29-32** | **47-50** | **33-39** | **30-34** |
| **10 very high** | **53<** | **39<** | **33<** | **51<** | **40<** | **35<** |

*Source*: Own elaboration based on Bielawska-Batorowicz’s (2004) Polish adaptation of the Menopause Symptom List by Janette M. Perz
